# Construction of an integrated genetic linkage map for the A genome of *Brassica napus *using SSR markers derived from sequenced BACs in *B. rapa*

**DOI:** 10.1186/1471-2164-11-594

**Published:** 2010-10-22

**Authors:** Jinsong Xu, Xiaoju Qian, Xiaofeng Wang, Ruiyuan Li, Xiaomao Cheng, Yuan Yang, Jie Fu, Shunchang Zhang, Graham J King, Jiangsheng Wu, Kede Liu

**Affiliations:** 1 National Key Laboratory of Crop Genetic Improvement and National Center of Plant Gene Research (Wuhan), Huazhong Agricultural University, Wuhan 430070, China; 2 Plant Science Department, Rothamsted Research, Harpenden, AL5 2JQ, UK

## Abstract

**Background:**

The Multinational *Brassica rapa *Genome Sequencing Project (BrGSP) has developed valuable genomic resources, including BAC libraries, BAC-end sequences, genetic and physical maps, and seed BAC sequences for *Brassica rapa*. An integrated linkage map between the amphidiploid *B. napus *and diploid *B. rapa *will facilitate the rapid transfer of these valuable resources from *B. rapa *to *B. napus *(Oilseed rape, Canola).

**Results:**

In this study, we identified over 23,000 simple sequence repeats (SSRs) from 536 sequenced BACs. 890 SSR markers (designated as BrGMS) were developed and used for the construction of an integrated linkage map for the A genome in *B. rapa *and *B. napus*. Two hundred and nineteen BrGMS markers were integrated to an existing *B. napus *linkage map (BnaNZDH). Among these mapped BrGMS markers, 168 were only distributed on the A genome linkage groups (LGs), 18 distrubuted both on the A and C genome LGs, and 33 only distributed on the C genome LGs. Most of the A genome LGs in *B. napus *were collinear with the homoeologous LGs in *B. rapa*, although minor inversions or rearrangements occurred on A2 and A9. The mapping of these BAC-specific SSR markers enabled assignment of 161 sequenced *B. rapa *BACs, as well as the associated BAC contigs to the A genome LGs of *B. napus*.

**Conclusion:**

The genetic mapping of SSR markers derived from sequenced BACs in *B. rapa *enabled direct links to be established between the *B. napus *linkage map and a *B. rapa *physical map, and thus the assignment of *B. rapa *BACs and the associated BAC contigs to the *B. napus *linkage map. This integrated genetic linkage map will facilitate exploitation of the *B. rapa *annotated genomic resources for gene tagging and map-based cloning in *B. napus*, and for comparative analysis of the A genome within *Brassica *species.

## Background

*Brassica *species are one of the most important crop groups in terms of cultivated acreage, contribution to human and animal diets, and economic value. Of the six cultivated *Brassica *species, *B. napus*, *B. rapa*, *B. juncea*, and *B. carinata *provide about 12% of worldwide edible vegetable oil supplies [1]. They also provide many of the vegetables in our daily diet such as cauliflower, broccoli, cabbage, kohlrabi, kale (*B. oleracea*) and turnip, Pak-choi and Chinese cabbage (*B. rapa*) [2]. *Brassica *species are also a valuable source of dietary fiber, vitamin C and other anticancer compounds such as glucosinolates. In addition, rapeseed oil has been used as a biofuel, a desirable alternative for fossil oil worldwide.

*Brassica *species are closely related to the model plant *Arabidopsis thaliana*, and provide an opportunity to study genome rearrangements associated with polyploidization. Comparative mapping using molecular markers has already revealed extensive synteny between *Brassica *and *Arabidopsis *[3,4]. The genomes of diploid *Brassica *species and *Arabidopsis *diverged 14.5 to 20.4 million years ago (MYA) from a common ancestor [5-7]. Subsequent chromosomal rearrangements including segmental duplications or deletions and extensive interspersed gene loss or gain events since divergence from the common ancestor have resulted in the present diploid *Brassica *species [6,8], *B. rapa *(2n = 20, AA), *B. nigra *(2n = 16, BB) and *B. oleracea *(2n = 18, CC). The three amphidiploid species, *B. juncea *(2n = 36, AABB), *B. napus *(2n = 38, AACC), and *B. carinata *(2n = 34, BBCC) originated from relatively recent interspecific hybridizations among the three diploid species, most likely during human cultivation of the diploid crops [9]. The genome relationships between these *Brassica *species is commonly known as the triangle of U [10].

The importance of *Brassica *species to the world economy and human health, and their potential value as models for studying genome changes associated with polyploidization, have promoted an international effort to sequence the complete set of *Brassica *genomes [7,11,12]. *B. rapa *ssp. *pekinensis*, which has the smallest genome [13], was selected as the representative for *Brassica *A genome sequencing by the Multinational *Brassica *Genome Project (MBGP) http://www.brassica.info[7], with the original aim of establishing the complete sequence of this genome using a BAC-by-BAC strategy. The BrGSP developed various genomic resources, including mapping populations, DNA libraries and DNA sequences. Three reference linkage maps, derived from the BraCKDH [14], BraJWF3P [15], and BraVCS-DH http://www.brassica-rapa.org populations, were constructed and had been served as backbones for anchoring BACs and BAC contigs for chromosome-based genome sequencing in *B. rapa*. Three BAC libraries covering approximately 36 genome equivalents of *B. rapa *were constructed using restriction enzymes *Hind*III, *BamH*I and *Sau3A*I. A total of 200,017 BAC-end sequences (BESs) were then generated from these BAC libraries. As of August 7, 2007, a stringent Build 2 of the physical map was released that contained 1,428 contigs with an average length of 512 kb, covering an estimated 717 Mb equivalent to 1.3 × of the *B. rapa *genome http://www.brassica-rapa.org[16]. The physical map based on fingerprint analysis was integrated to the genetic map by STS and SSR markers and chromosome fluorescent in situ hybridization (FISH) analysis, which enabled the positioning of 242 gene-rich contigs to specific locations on the 10 LGs [16]. Based on the physical map of *B. rapa *and the *in silico *comparative map of BAC-ends onto *Arabidopsis *chromosomes, 629 'seed' BACs spanning 86 Mb of *Arabidopsis *euchromatic regions were selected, distributed throughout the *B. rapa *genome http://www.brassica-rapa.org[7]. These anchored BAC clones were sequenced to provide starting points ('seeds') from which to continue the whole genome sequencing http://www.brassica-rapa.org.

SSRs, or microsatellites, are tandem repeats of 1-6 nucleotide sequence motifs flanked by unique sequences [17]. SSRs have become desirable molecular markers for gene tagging, germplasm evaluation, molecular-assisted selection and comparative mapping [18] because they have several advantages over other DNA markers, including being co-dominant, highly polymorphic, abundant, and distributed throughout the genome [19]. The DNA sequence information generated from BAC-ends and anchored BACs in *B. rapa *by the BrGSP (e.g. http://www.brassica.bbsrc.ac.uk; http://www.brassica-rapa.org) provided an opportunity to evaluate the abundance and relative distribution of these SSRs in the whole genome [20,21], and an excellent opportunity to develop a large number of markers with known map positions to establish direct links between genetic, physical, and sequence-based maps of the *Brassica *crop species.

To utilize the invaluable genomic resources developed in *B. rapa *for genome analysis and genetic improvement in *B. napus *and other cultivated *Brassica *species, it is necessary to integrate the genetic and physical maps to the existing *B. napus *genetic maps [22,23]. The objectives of this study were to develop SSR markers from publicly available sequenced BACs in *B. rapa*, to integrate these markers to the existing *B. napus *linkage map [22], and to anchor these sequenced BACs and their associated BAC contigs to the A genome in *B. napus*. Here we report the identification and characterization of SSRs derived from sequenced BACs in *B. rapa *and the construction of an integrated genetic map in the A genomes of *B. napus*.

## Results

### Characterization of microsatellites in sequenced BACs of *B. rapa*

As this study started, a total of 536 seed BACs had been sequenced to phase II or phase III by the BrGSP and deposited into NCBI-GenBank http://www.brassica.info/resource/sequencing/status.php. These had been used as starting points for the selection of BACs to extend contigs in two directions with minimum overlap, based on matches to BAC-end sequences [7]. As indicated at http://www.brassica-rapa.org, 232 of these BACs had been completely sequenced (phase III) and the remainders were sequenced to phase II (i.e. fully oriented and ordered sequence with some small gaps). Of these, 482 had provisionally been assigned to specific chromosomes of *B. rapa*, and 54 unassigned to any specific chromosome. Considerably more BACs had been sequenced for chromosomes A3 and A9 (153 and 85 respectively), which had been allocated to the Korea Brassica Genome Project (KBGP). Chromosome A4, which is the shortest LGs in many linkage maps [14,22,24], had the least number of sequenced BACs (22). Twenty seven to forty four sequenced BACs were assigned to each of the remaining seven chromosomes. The total length of the sequenced BACs was 63.5 Mb, representing a coverage of 11.5% of the A genome and corresponding to 86 Mb of the euchromatic regions of the *Arabidopsis *genome http://www.brassica-rapa.org[7].

A total of 23,192 SSRs were identified within the 536 sequenced BACs corresponding to a mean density of one in every 2.74 kb. Table 1 summarized the frequencies of major SSRs identified within the sequenced BACs. Tri-nucleotide repeats were the most abundant (37.61%), followed by di- (36.21%), tetra- (15.59%) and penta-nucleotides (10.59%) (Table 1). The most abundant repeat motif was (AT)_n _(20.34%), followed by (AG)_n _(12.92%), (AAG)_n _(11.26%), (AAT)_n _(6.67%) and (AAAT)_n _(5.70%), reflecting the AT-rich nature of the *B. rapa *genome [21]. In di-nucleotide repeats, the most frequent motif was (AT)_n _(20.34%), followed by (AG)_n _(12.92%) and (AC)_n _(2.94%) motifs. The (GC)_n _motif was very rare (0.01%) (Table 1). Ten different tri-nucleotide repeats were observed, with the (AAG)_n _motif (11.26%) being the most common, followed by the (AAT)_n _(6.67%) and (ATC)_n _(5.43%) (Table 1). The GC-rich motifs (10.32%) were much less than the AT-rich motifs (27.29%). Thirty two tetra-nucleotide repeats and 85 penta-nucleotide repeats were observed, respectively. (AAAN)_n _(10.15%) and (AAAAN)_n _(4.23%), especially (AAAT)_n _(5.70%) and (AAAAT)_n _(2.95%), were the most frequent motifs for the tetra- and penta-nucleotide repeats. The other tetra- and penta-nucleotide repeats were very rare. The SSR repeat length ranged from 12 to 214 bp, with the di-nucleotide repeats showing the greatest length variation and average repeat length (Table 1), which was consistent with the length distributions in many other eukaryotic genomes [25].

**Table 1:** Distribution of the major SSR types identified from the seed BACs in *B. rapa*

Motifs	Number (%)	Range (bp)	Average length (bp)
Dinucleotide	8398 (36.21)	12-214	19.13
AT	4718 (20.34)	12-210	19.74
AG	2997 (12.92)	12-214	19.08
AC	680 (2.94)	12-74	14.94
CG	2 (0.01)	12-16	14.00
Tri-nucleotide	8722 (37.61)	12-102	13.79
AAG	2612 (11.26)	12-93	13.91
AAT	1547 (6.67)	12-102	14.00
ATC	1246 (5.43)	12-51	13.85
AAC	929 (4.00)	12-42	13.77
AGG	889 (3.83)	12-39	13.75
ACC	608 (2.62)	12-27	13.69
AGC	339 (1.46)	12-27	13.33
ACG	194 (0.83)	12-30	13.13
ACT	185 (0.80)	12-24	13.23
CCG	173 (0.71)	12-24	13.20
Tetranucleotide	3616 (15.59)	12-52	13.26
AAAT	1323 (5.70)	12-36	13.18
AAAG	483 (2.08)	12-40	13.57
AAAC	455 (2.37)	12-28	13.44
Others	1355(5.44)	12-52	13.16
Pentanucleotide	2457 (10.59)	15-45	14.56
AAAAT	685 (2.95)	15-30	14.86
AAAAC	239 (1.03)	15-30	15.84
AAAAG	171 (0.73)	15-20	15.53
Others	1362 (5.88)	15-45	14.07
Total	23192 (100.00)	12-214	14.16

### Development of microsatellite markers from sequenced BACs

A total of 890 primer pairs, designated as BrGMSs, were designed from the sequenced BACs to amplify the simple sequence repeats. At least one SSR marker was developed from each BAC. The priority was given to the di- and tri-nucleotide repeats because they had higher success rates of amplification and higher levels of polymorphism [22]. Among these markers, the largest proportion was (AG)_n_, followed by (AT)_n_, (AAG)_n _and (AC)_n _motifs (Table 2). No (CG)_n_, (CCG)_n _and (ACT)_n _motifs were included because they are very rare in the BAC sequences. Table 2 summarized the features of the new developed BrGMS markers.

**Table 2:** Percentage of polymorphic markers for successfully amplified primer pairs

Motifs	Primers designed	Amplified primers^a ^(%)	Polymorphic primers^b ^(%)
Di-nucleotides	666	591(88.74)	339(50.90)
AG	338	306(90.53)	193(57.10)
AT	294	254(86.39)	128(43.54)
AC	34	31(91.18)	18(52.94)
Tri-nucleotides	164	150(91.46)	92(58.10)
AAG	61	56(91.80)	37(60.66)
ATG	35	33(94.29)	20(57.14)
AGG	18	17(94.44)	8(44.44)
AAT	21	19(90.48)	11(52.38)
AAC	16	14(87.50)	8(50.00)
GC-rich TNRs	13	11(84.62)	8(61.54)
Tetra-nucleotides	30	29(96.67)	16(53.33)
Penta-nucleotides	30	24(80.00)	13(43.33)
Total	890	794(89.21)	460(51.69)

^a ^Percentage of successfully amplified SSRs per designed primer pairs

^b ^Percentage of polymorphic markers per successfully amplified primer pairs based on the screening of six *B. napus *lines.

The 890 BrGMS markers were tested for amplification using a panel of six rapeseed varieties that had been used in previous study [22]. Of these, 794 (89.21%) markers successfully amplified at least one PCR product from the *B. napus *genome, and 460 (51.7%) were polymorphic among the six varieties. A downloadable file including the marker names, primer sequences, the source BACs and chromosomes, the amplification and scorability of the markers was available as Additional file 1.

### Integration of the BrGMS markers to the *B. napus *linkage map

**Figure 1:**
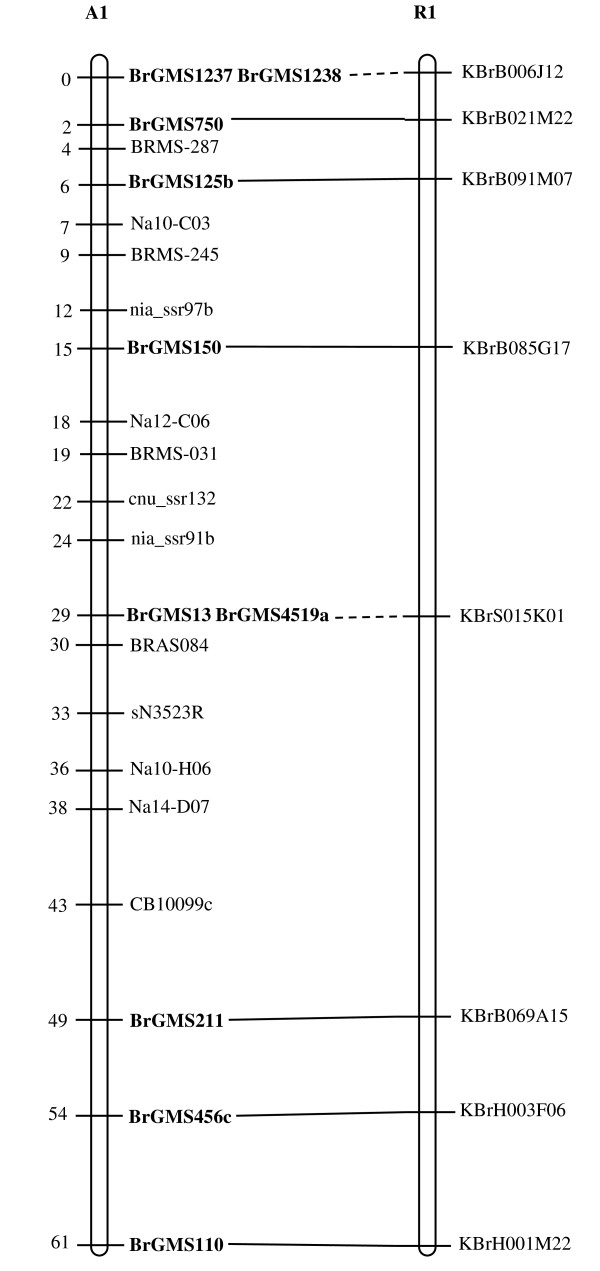
**Integration of A1 in *B. rapa *and *B. napus *using SSR markers derived from the same sequenced BACs**. Cumulative recombination distances in cM are shown to the left and marker loci to the right of the linkage group. SSR markers developed from sequenced BACs are indicated in boldface. The correspondence between the SSR markers and the BAC clones is given in Additional file 1. The map was constructed using 88 DH plants derived from No. 2127 and ZY821 using BrGMS (*boldface*) markers and public SSRs as anchors [22]. Markers from the same BAC are connected by lines to indicate the collinearity between *B. napus *and *B. rapa*. The *B. rapa *BraJWF3P and BraVCS-DH linkage maps were redrawn from www.brassica-rapa.org. The dotted lines indicated the assignments of previously unassigned BACs.

**Figure 2:**
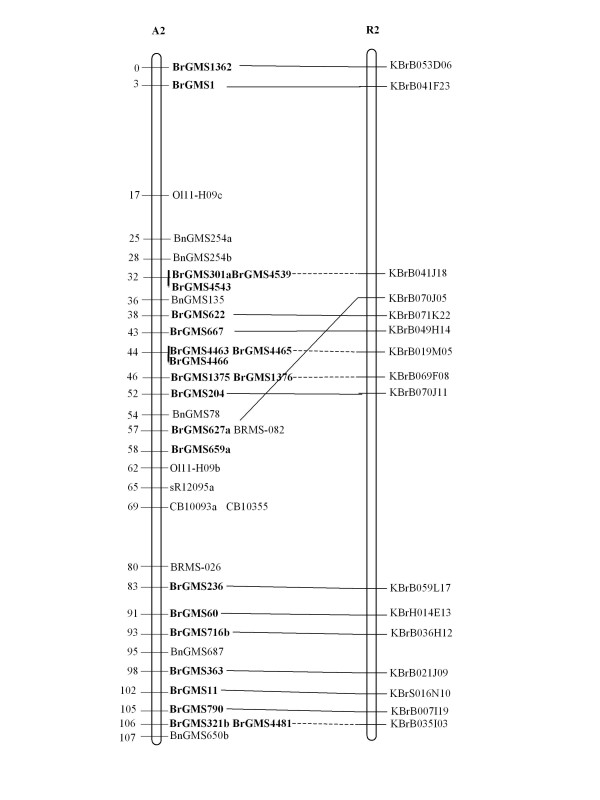
**Integration of A2 in *B. rapa *and *B. napus *using SSR markers derived from the same sequenced BACs**. Cumulative recombination distances in cM are shown to the left and marker loci to the right of the linkage group. SSR markers developed from sequenced BACs are indicated in boldface. The correspondence between the SSR markers and the BAC clones is given in Additional file 1. The map was constructed using 88 DH plants derived from No. 2127 and ZY821 using BrGMS (*boldface*) markers and public SSRs as anchors [22]. Markers from the same BAC are connected by lines to indicate the collinearity between *B. napus *and *B. rapa*. The *B. rapa *BraJWF3P and BraVCS-DH linkage maps were redrawn from http://www.brassica-rapa.org. The dotted lines indicated the assignments of previously unassigned BACs.

**Figure 3:**
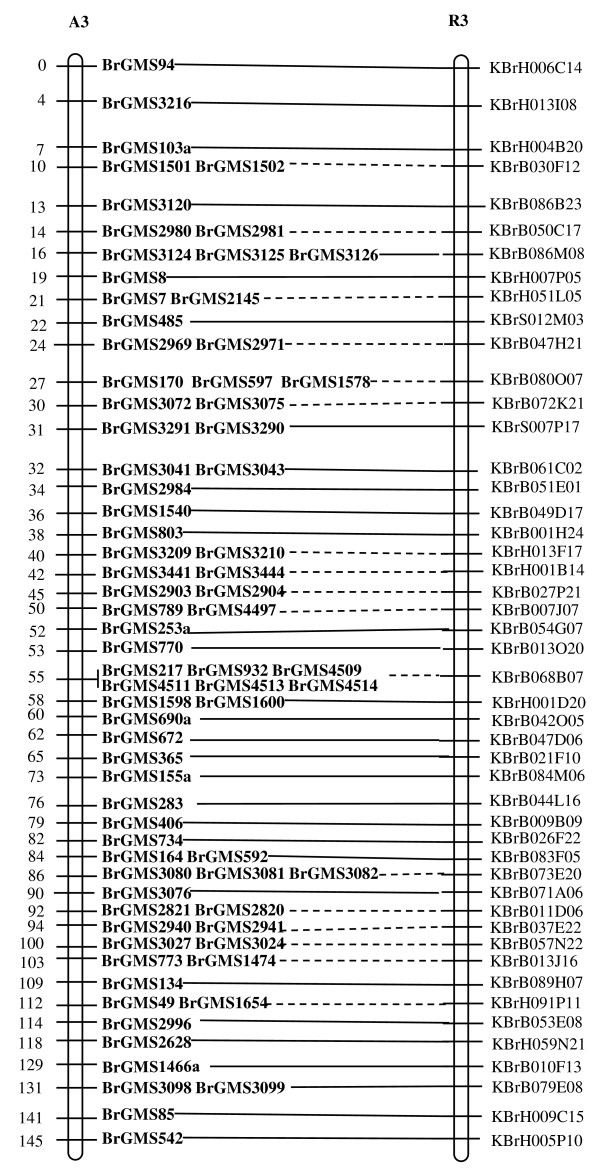
**Integration of A3 in *B. rapa *and *B. napus *using SSR markers derived from the same sequenced BACs**. Cumulative recombination distances in cM are shown to the left and marker loci to the right of the linkage group. SSR markers developed from sequenced BACs are indicated in boldface. The correspondence between the SSR markers and the BAC clones is given in Additional file 1. The map was constructed using 88 DH plants derived from No. 2127 and ZY821 using BrGMS (*boldface*) markers and public SSRs as anchors [22]. Markers from the same BAC are connected by lines to indicate the collinearity between *B. napus *and *B. rapa*. The *B. rapa *BraJWF3P and BraVCS-DH linkage maps were redrawn from http://www.brassica-rapa.org. The dotted lines indicated the assignments of previously unassigned BACs.

**Figure 4:**
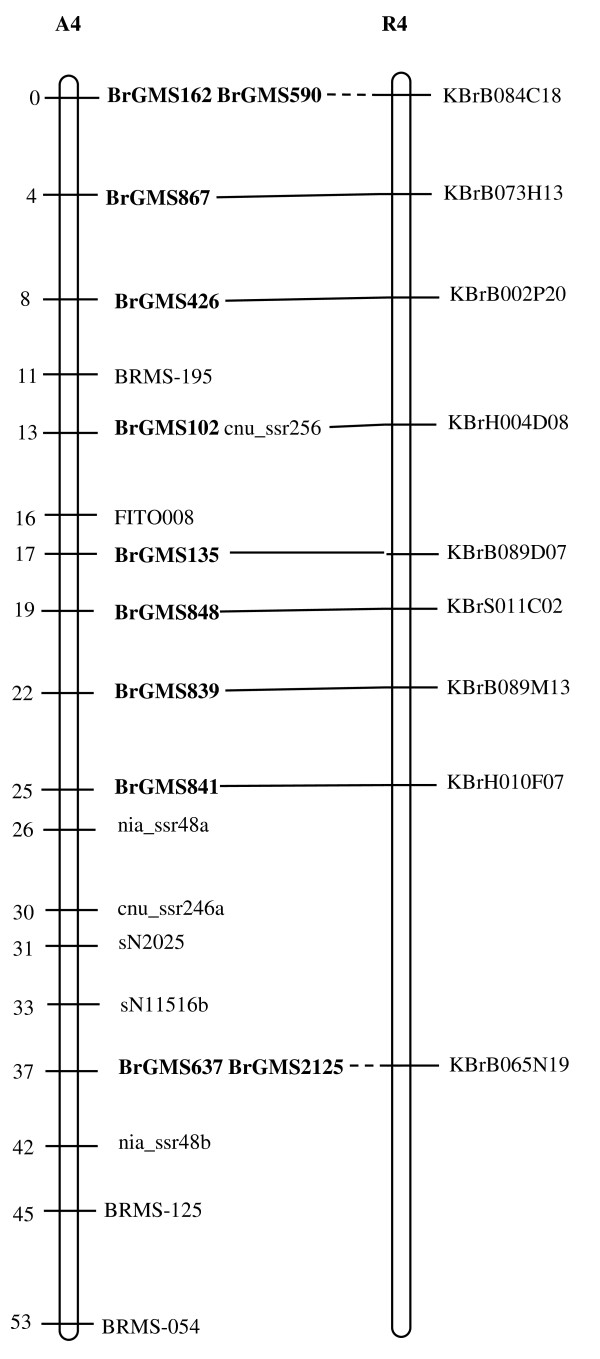
**Integration of A4 in *B. rapa *and *B. napus *using SSR markers derived from the same sequenced BACs**. Cumulative recombination distances in cM are shown to the left and marker loci to the right of the linkage group. SSR markers developed from sequenced BACs are indicated in boldface. The correspondence between the SSR markers and the BAC clones is given in Additional file 1. The map was constructed using 88 DH plants derived from No. 2127 and ZY821 using BrGMS (*boldface*) markers and public SSRs as anchors [22]. Markers from the same BAC are connected by lines to indicate the collinearity between *B. napus *and *B. rapa*. The *B. rapa *BraJWF3P and BraVCS-DH linkage maps were redrawn from http://www.brassica-rapa.org. The dotted lines indicated the assignments of previously unassigned BACs.

**Figure 5:**
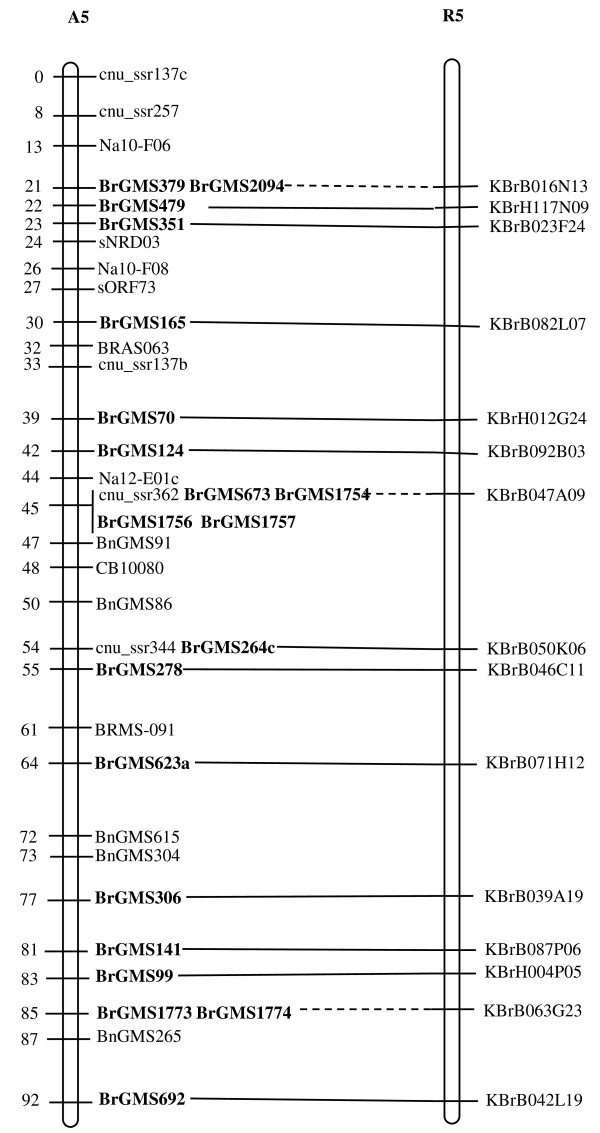
**Integration of A5 in *B. rapa *and *B. napus *using SSR markers derived from the same sequenced BACs**. Cumulative recombination distances in cM are shown to the left and marker loci to the right of the linkage group. SSR markers developed from sequenced BACs are indicated in boldface. The correspondence between the SSR markers and the BAC clones is given in Additional file 1. The map was constructed using 88 DH plants derived from No. 2127 and ZY821 using BrGMS (*boldface*) markers and public SSRs as anchors [22]. Markers from the same BAC are connected by lines to indicate the collinearity between *B. napus *and *B. rapa*. The *B. rapa *BraJWF3P and BraVCS-DH linkage maps were redrawn from http://www.brassica-rapa.org. The dotted lines indicated the assignments of previously unassigned BACs.

**Figure 6:**
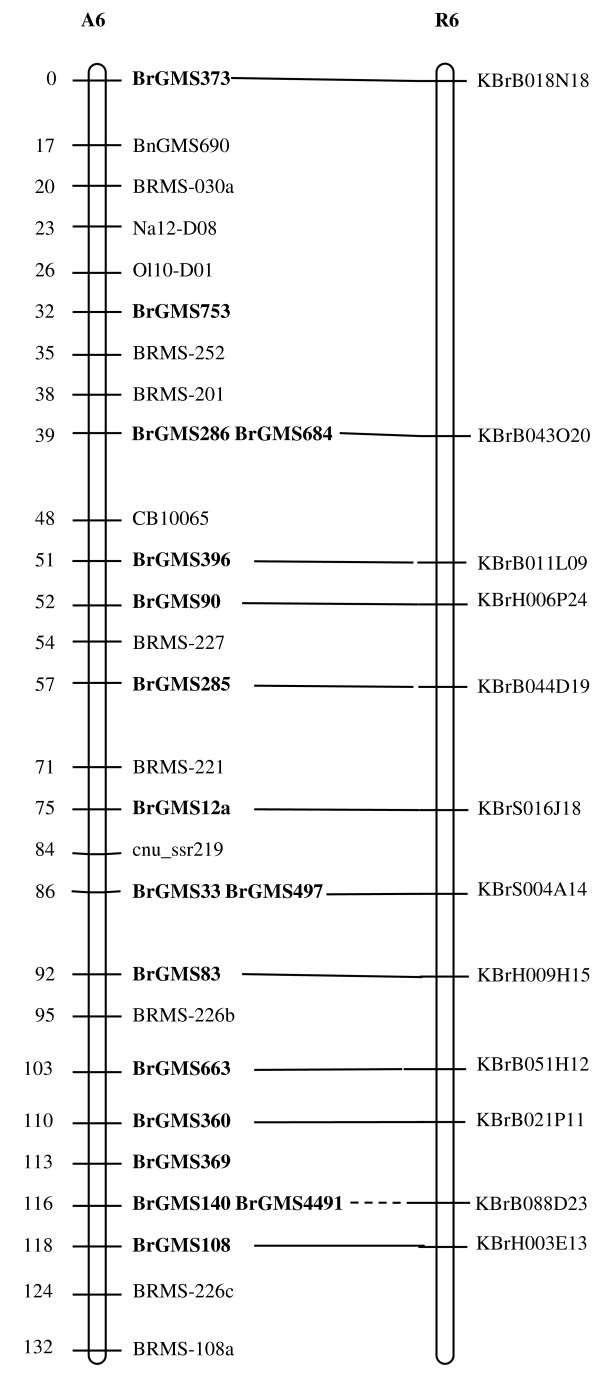
**Integration of A6 in *B. rapa *and *B. napus *using SSR markers derived from the same sequenced BACs**. Cumulative recombination distances in cM are shown to the left and marker loci to the right of the linkage group. SSR markers developed from sequenced BACs are indicated in boldface. The correspondence between the SSR markers and the BAC clones is given in Additional file 1. The map was constructed using 88 DH plants derived from No. 2127 and ZY821 using BrGMS (*boldface*) markers and public SSRs as anchors [22]. Markers from the same BAC are connected by lines to indicate the collinearity between *B. napus *and *B. rapa*. The *B. rapa *BraJWF3P and BraVCS-DH linkage maps were redrawn from http://www.brassica-rapa.org. The dotted lines indicated the assignments of previously unassigned BACs.

**Figure 7:**
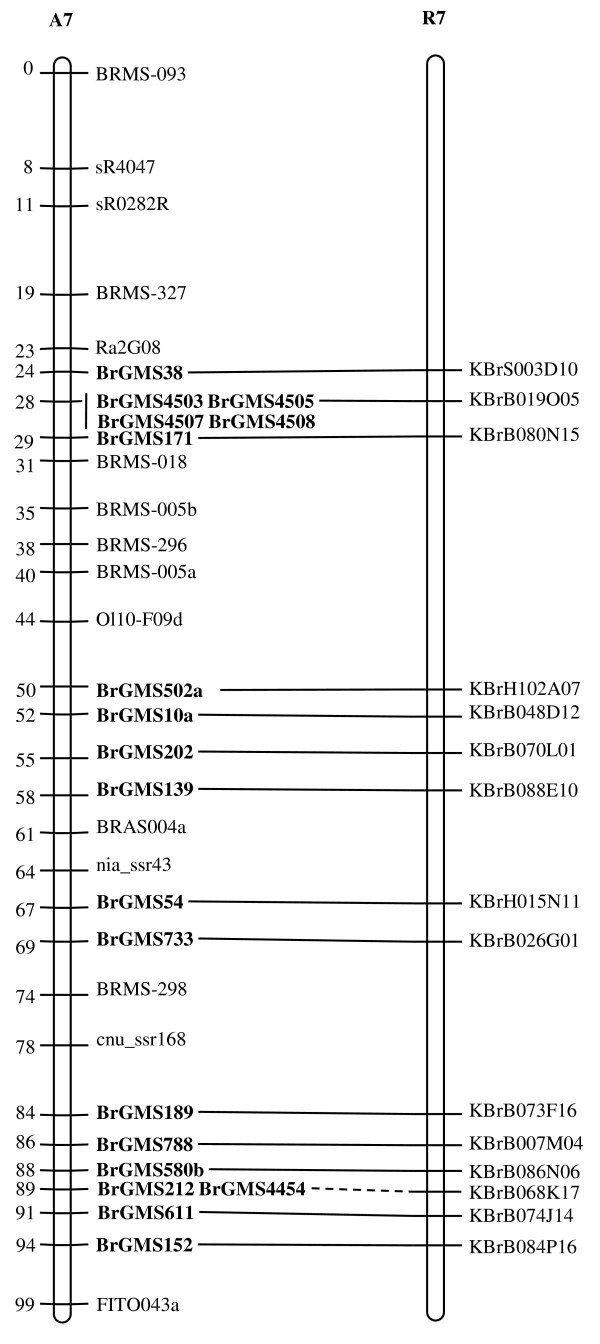
**Integration of A7 in *B. rapa *and *B. napus *using SSR markers derived from the same sequenced BACs**. Cumulative recombination distances in cM are shown to the left and marker loci to the right of the linkage group. SSR markers developed from sequenced BACs are indicated in boldface. The correspondence between the SSR markers and the BAC clones is given in Additional file 1. The map was constructed using 88 DH plants derived from No. 2127 and ZY821 using BrGMS (*boldface*) markers and public SSRs as anchors [22]. Markers from the same BAC are connected by lines to indicate the collinearity between *B. napus *and *B. rapa*. The *B. rapa *BraJWF3P and BraVCS-DH linkage maps were redrawn from http://www.brassica-rapa.org. The dotted lines indicated the assignments of previously unassigned BACs.

**Figure 8:**
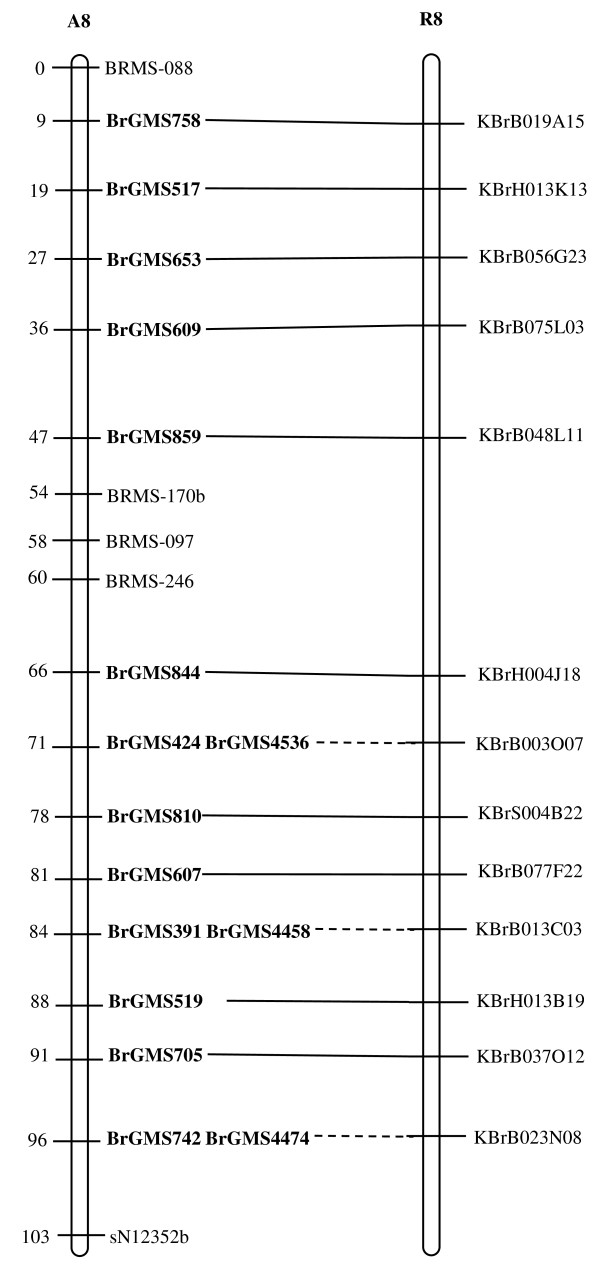
**Integration of A8 in *B. rapa *and *B. napus *using SSR markers derived from the same sequenced BACs**. Cumulative recombination distances in cM are shown to the left and marker loci to the right of the linkage group. SSR markers developed from sequenced BACs are indicated in boldface. The correspondence between the SSR markers and the BAC clones is given in Additional file 1. The map was constructed using 88 DH plants derived from No. 2127 and ZY821 using BrGMS (*boldface*) markers and public SSRs as anchors [22]. Markers from the same BAC are connected by lines to indicate the collinearity between *B. napus *and *B. rapa*. The *B. rapa *BraJWF3P and BraVCS-DH linkage maps were redrawn from http://www.brassica-rapa.org. The dotted lines indicated the assignments of previously unassigned BACs.

Of the polymorphic BrGMS markers, 219 that showed polymorphism between the DH line 'No.2127' and the inbred line 'ZY821' were utilized to genotype the BnaNZDH population. Among these markers, 201 detected only one polymorphic locus, 18 detected two polymorphic loci. Of the 201 markers with a single polymorphic locus, 168 were mapped to the A1 to A10 LGs of the A genome and 33 to the C1 to C9 LGs of the C genome. The 18 markers with two polymorphic loci were mapped to both the A and C genomes, which was often observed in genetic maps of *B. napus *due to the high level of sequence homology between the A and C genomes [4,23]. In total, 186 BrGMS loci were integrated to the A1 to A10 LGs of the existing genetic linkage map constructed using the same population [22]. The A genome LGs covered a total length of 1013.4 cM, with an average distance of 3.34 cM between adjacent loci (Figure 1, 2, 3, 4, 5, 6, 7, 8, 9 &10). The BrGMS loci on A1 to A10 were evenly distributed.

**Figure 9:**
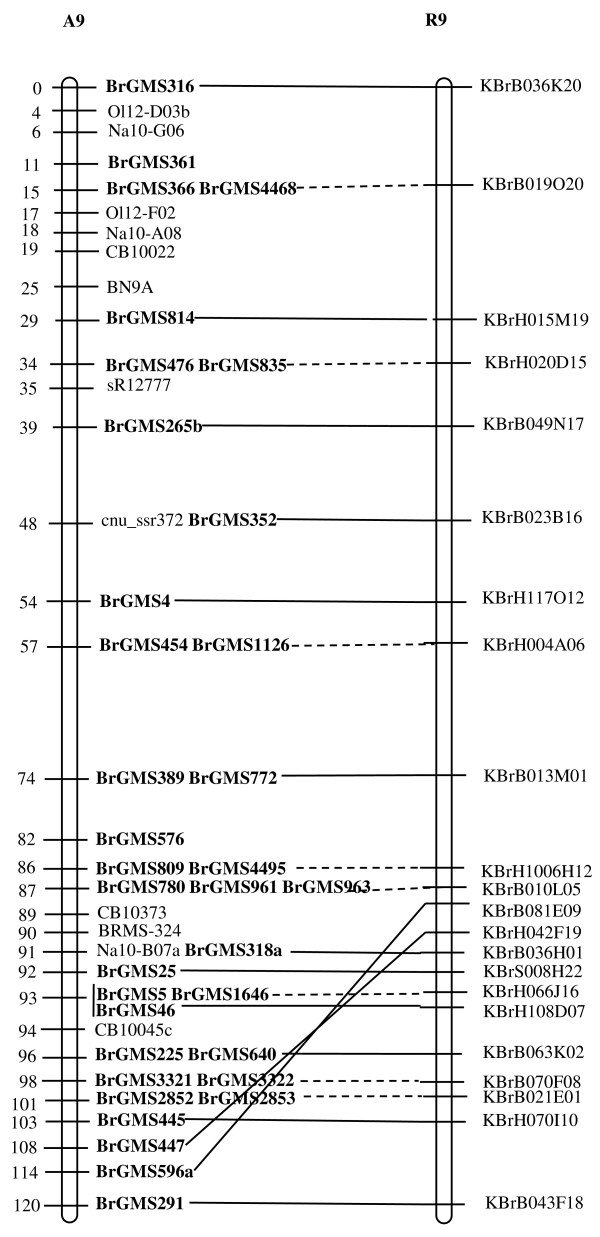
**Integration of A9 in *B. rapa *and *B. napus *using SSR markers derived from the same sequenced BACs**. Cumulative recombination distances in cM are shown to the left and marker loci to the right of the linkage group. SSR markers developed from sequenced BACs are indicated in boldface. The correspondence between the SSR markers and the BAC clones is given in Additional file 1. The map was constructed using 88 DH plants derived from No. 2127 and ZY821 using BrGMS (*boldface*) markers and public SSRs as anchors [22]. Markers from the same BAC are connected by lines to indicate the collinearity between *B. napus *and *B. rapa*. The *B. rapa *BraJWF3P and BraVCS-DH linkage maps were redrawn from http://www.brassica-rapa.org. The dotted lines indicated the assignments of previously unassigned BACs.

**Figure 10:**
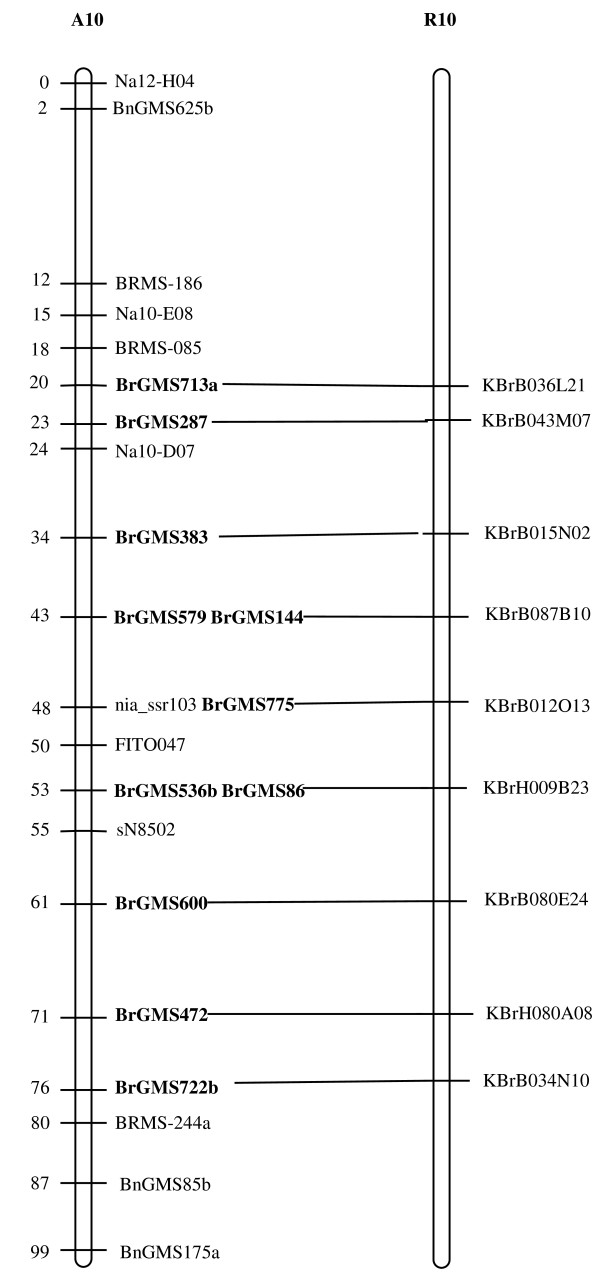
**Integration of A10 in *B. rapa *and *B. napus *using SSR markers derived from the same sequenced BACs**. Cumulative recombination distances in cM are shown to the left and marker loci to the right of the linkage group. SSR markers developed from sequenced BACs are indicated in boldface. The correspondence between the SSR markers and the BAC clones is given in Additional file 1. The map was constructed using 88 DH plants derived from No. 2127 and ZY821 using BrGMS (*boldface*) markers and public SSRs as anchors [22]. Markers from the same BAC are connected by lines to indicate the collinearity between *B. napus *and *B. rapa*. The *B. rapa *BraJWF3P and BraVCS-DH linkage maps were redrawn from http://www.brassica-rapa.org.

The 186 BrGMS loci were derived from 161 sequenced BACs. Of these loci, 147 were derived from BACs that had been assigned to specific chromosomes on the *B. rapa *physical map, while 39 loci were derived from 27 previously unassigned BACs. Among these 161 BACs, 21 BACs each had two BrGMS loci and two BACs each had three loci mapped to the A genome in *B. napus*, and the remaining 138 each had one locus mapped to the A genome. Of the 186 loci, 18 detected null alleles in one parent, which was also observed in previous research in *Brassica *[26,27]. No.2127 had 13 null alleles, while ZY821 had five null alleles.

### Comparative analysis between the *B. rapa *and *B. napus *linkage maps

Three reference linkage maps, the BraCKDH, BraJWF3P and BraVCS-DH, were previously developed to assign BACs and BAC contigs to chromosomes for genome sequencing in *B. rapa *http://www.brassica-rapa.org[14,15,28]. These maps contained many common markers derived from the same set of sequenced BACs as those used in this study for marker development. These sequenced BACs had been provisionally anchored to the *B. rapa *linkage map through genetic mapping of SSR or STS markers [28]. For ease of comparison, we constructed an integrated *B. rapa *linkage map based on the common markers that were shared between two or more maps or different markers derived from the same BAC and had similar map positions on two or three reference linkage maps. The integrated linkage map was constructed merely based on the published map positions and marker orders on the three reference linkage maps. The orientation of LGs for the three *B. rapa *reference maps were not consistent, for the A genome the agreed orientation were consistent with the BraCKDH map [14,28], so we finished the integrated *B. rapa *linkage map based on the orientation of LGs in BraCKDH map [14,28]. We referred to the SSR markers on the three *B. rapa *reference maps and the subsequent integrated linkage map as 'KBGP markers', to distinguish them from those developed in this study. The *B. napus *linkage map constructed in this study was compared with the integrated *B. rapa *linkage map based on SSR markers developed from the same BACs. All LGs in *B. napus *were collinear with their corresponding homologues in *B. rapa *(Figure 1, 2, 3, 3, 4, 5, 6, 7, 8, 9 &10), apart from A2 and A9, where single minor intra-chromosomal inversions or rearrangements involved one or two markers [4,15].

### Assignment of the *B. rapa *BACs and BAC contigs to the *B. napus *linkage map

The mapped SSR markers were then used to construct a map to integrate the A genome map for *B. napus *with that of *B. rapa *genetic maps. The sequenced BACs were assigned to the A genome LGs based on map locations of the KBGP and/or BrGMS markers on the *B. rapa *and *B. napus *linkage maps. Our criterion for assigning a BAC to a map location required at least two markers derived from the same BAC being mapped to the same position or adjacent position. Due to the polyploid nature of the *Brassica *A genome, it is possible that a BAC could be incorrectly mapped to a paralogous position, if by chance independent evolution of the duplicated regions in the A genomes of *B. rapa *and *B. napus *had led to loss of a SSR locus in one but not the other. Within *B. rapa *the only unambiguous assignment was achieved either where there has been allele matching between the BAC sequence and the same genotype (Chiifu-401) and/or there has been single signal derived from chromosome FISH of the BAC [28]. For the 134 BACs that had been previously anchored to the *B. rapa *linkage maps, we carefully compared the map locations of the KBGP markers on the *B. rapa *linkage maps with those of the BrGMS markers derived from the same BAC on the *B. napus *linkage map, as well as with the anchoring of their source BAC on the *B. rapa *physical map. If the KBGP and BrGMS markers derived from the same BAC were mapped to similar positions on the same LGs on the *B. rapa *and *B. napus *linkage maps, and their source BAC was assigned to the same LGs on the *B. rapa *physical map, the BAC and its associated BAC contig were assigned to the *B. napus *genetic map. Where this was not the case, additional SSR markers were developed from the BAC and mapped to confirm its assignment to the *B. napus *linkage map. Based on the above criteria, 125 BACs and their associated BAC contigs could be unequivocally assigned to corresponding *B. napus *chromosomes (Figure 1, 2, 3, 4, 5, 6, 7, 8, 9 &10 and Additional file 2). For example, marker KS50880 derived from KBrB071K22 was mapped to A2 on the BraJWF3P map and its associated BAC contig (contig31) was assigned to chromosome A2 on the physical map [16]. BrGMS622 derived from the same BAC was mapped to the similar position (within 1 cM) on A2 on the *B. napus *linkage map. Therefore we could unequivocally assign KBrB071K22 and its associated contig31 to A2 on the *B. napus *genetic map (Figure 1, 2, 3, 4, 5, 6, 7, 8, 9 &10 and Additional file 2). Nine BACs could not be directly assigned to the *B. napus *linkage map due to conflicts between the *B. rapa *and *B. napus *linkage maps or between the genetic and physical maps. Therefore we assigned these BACs to the *B. napus *linkage map by mapping two or more additional SSR markers derived from each BAC.

The *B. napus *linkage map also contained 39 BrGMS loci derived from 27 previously unassigned BACs. Ten of the BACs each had at least two markers located at similar positions on the *B. napus *linkage map, and so these 10 BACs were then assigned to the *B. napus *LGs (Figure 1, 2, 3, 4, 5, 6, 7, 8, 9 &10 and Additional file 3). The remaining 17 BACs each had one SSR marker mapped to the *B. napus *linkage map. To anchor these 17 BACs unambiguously to specific *B. napus *LGs, five to 10 additional SSR markers were developed from each BAC and used to screen polymorphisms between the two parents. Polymorphic SSR markers were integrated to the *B. napus *linkage map. Sixteen BACs (Additional file 3) each had two or three SSR markers mapped to the same positions on the *B. napus *linkage map, and thus could be assigned to specific LGs. Another BAC, KBrB019M05, had four mapped SSR markers, with three markers (BrGMS4463, BrGMS4465 and BrGMS4466) being mapped to the same position on A2 and the other one (BrGMS369) to A6, a paralogous segment of A2 [15], suggesting that KBrB019M05 should be assigned to A2. Therefore, the mapping of SSR markers enabled the assignment of these BACs and their associated BAC contigs to specific LGs.

In the above analysis, we unequivocally assigned a total of 161 BACs to the *B. napus *linkage map, and thus constructed an integrated genetic map for the A genome of *B. napus*.

### Validation of BAC assignment

To test the reliability of the assignment of BACs on the *B. rapa *physical map to the *B. napus *genetic map, we compared the map positions of markers derived from the same BACs. Fourteen BACs each had two SSR markers mapped on the *B. napus *linkage map (Additional file 4). The markers derived from the same BAC were mapped to the same positions on the *B. napus *linkage map (Figure 1, 2, 3, 4, 5, 6, 7, 8, 9 &10) and it was confirmed that the assignments of these BACs were correct.

We further checked the map positions of marker loci that were derived from different BACs that were present within the same contigs. There were 16 BAC contigs that each had two or three different constituent BACs assigned to the genetic and physical map (Additional file 4). All SSR markers derived from BACs in the same contig were mapped at adjacent positions on the corresponding LGs (Figure 1, 2, 3, 4, 5, 6, 7, 8, 9 &10 and Additional file 4). For example, BrGMS672 and BrGMS690 were derived from KBrB047D06 and KBrB042O05 respectively, within contig73 on A3, and were 3 cM apart on the *B. napus *linkage map (Figure 1, 2, 3, 4, 5, 6, 7, 8, 9 &10). These results also demonstrated that the assignment of BACs to the A genome of *B. napus *were reliable.

To further validate the integrated genetic map constructed in this study, we selected five BACs randomly from each of five contigs, to develop and map additional SSR markers (Additional file 4). The additional markers derived from each BAC co-segregate with the previously mapped SSR markers (Figure 1, 2, 3, 4, 5, 6, 7, 8, 9 &10 and Additional file 4). These results further indicated that the assignments of *B. rapa *BACs and BAC contigs to the *B. napus *linkage map were reliable.

## Discussion

### Characterization of SSRs in *B. rapa*

Previously, our view of the abundance of microsatellites in *Brassica *had largely been inferred from hybridization screening [29-32] and analysis of genome survey sequences (GSSs) such as BAC end sequences (BES) [21] and whole genome shotgun sequences [33]. In this study, we analyzed the frequency of microsatellites identified from 536 sequenced BACs, which represents a total length of 63.5 Mb of the *B. rapa *genome and corresponds to a coverage of the gene-rich euchromatic regions of the *Arabidopsis *genome that are distributed across all 10 LGs of the *B. rapa *genome [7]. The frequency of SSR occurrence (one every 2.74 kb) in the BAC sequences of *B. rapa *is similar to that in the completely sequenced PAC and BAC clones in rice (one every 2.7 kb) [34]. This frequency is much higher than that in BAC-end sequences of *B. rapa *(one per 4.7 kb) [21] and *B. napus *(one every 4.0 kb) [22], and in the whole genome shotgun sequences of *B. oleracea *(one every 4.0 kb) [33], indicating that the frequency of SSRs identified from GSSs of *B. rapa*, *B. oleracea *and *B. napus *underestimated the abundance of microsatellites in *Brassica *genomes. In these BAC sequences, tri-nucleotide repeats (37.61%) are the most frequent within the *B. rapa *genome, followed by di-nucleotide repeats (36.21%). Of the tri-nucleotide repeats, the motif (AAG)_n _is the most frequent, consistent with the pattern in BES of *B. rapa *[21]. Among di-nucleotide repeats, the ranking of motifs is (AT)_n _> (AG)_n _> (AC)_n _repeats. This distribution is in good agreement with that observed in the complete genomes of *Arabidopsis *[25] and rice [34]. This compares with the ranking of (AG)_n _> (AT)_n _> (AC)_n _in BAC-end sequences of *B. rapa *[21], as well as in whole genome shotgun sequences of *B. oleracea *[33]. This discrepancy might be due to the non-random distribution of these repeat motifs, the biases introduced in the process of library construction, and the difficulty of sequencing long stretches of self-complimentary (AT)_n _repeats which appeared in BAC ends or small-insert genomic libraries using the single pass sequencing strategy. These data led to the conclusion that the BAC sequences, rather than the GSSs, could be more representative of the actual occurrence and composition of SSRs in the complete genomes of *Brassica *species [34].

### Comparative mapping of the A genome in *B. rapa *and *B. napus*

SSR markers have previously been developed from various sources including small-insert genomic libraries [29], BAC ends [14,26], ESTs [35] and whole-genome shotgun sequences [33], and contributed to many genetic linkage maps using different mapping populations [22-24,36]. However, until now these maps have been difficult to align and integrate due to the lack of common markers. The three *B. rapa *reference maps included many common SSR markers developed from hundreds of sequenced BACs, and so an integrated linkage map was able to be constructed for *B. rapa *based on these common markers [15]http://www.brassica-rapa.org. Our 890 BrGMS markers were developed from the same set of sequenced BACs as used for the construction of the *B. rapa *reference maps. Common BACs were then used as bridges to align the A genome LGs in *B. rapa *and *B. napus*. As seen in Figure 1, 2, 3, 4, 5, 6, 7, 8, 9 &10, all the A genome LGs showed good collinearity between *B. rapa *and *B. napus*. These analyses suggested that the A genome is retained relatively intact in *B. napus *[37] despite extensive genome rearrangements such as insertions and deletions that have occurred following hybridization of the A and C genomes [38,39]. The gene order and content are conserved in the A genome of *B. napus *and *B. rapa *as revealed by comparative sequencing [39]. Comparative mapping [40] also showed that these LGs appear intact within *B. juncea*, the other amphidiploid of the triangle of U [10] that contains the A genome. The markers developed in this study will therefore be valuable for ongoing comparative analysis among *Brassica *species, especially within the A genomes.

### Integrated genetic and physical map in *B. napus*

Map integration through the use of common markers is a powerful tool for anchoring BACs and BAC contigs to genetic and physical maps [16]. The mapping of a single marker can often result in mis-assignment of BACs due to highly conserved intra- and inter-chromosomal duplications, or recently evolved gene paralogs that have distinct locations in the triplicated *B. rapa *genome [16]. In *B. rapa*, anchoring of sequenced BAC clones to linkage maps was achieved by matching alleles amplified from the genomic DNA of Chiifu-401 and BAC clones derived from the same genotype (Chiifu-401) [28], which is the only way to assign BACs uniquivocally to a genetic map, especially where there are extensive duplications and triplications, as in *B. rapa *genome. In this study, we assigned BACs to the *B. napus *genetic map through mapping of at least two markers derived from the same BAC. Single BACs were assigned only when two or more markers derived from the same BAC were mapped to the same or adjacent positions. Using this strategy, we assigned 161 sequenced BACs and their associated BAC contigs to the *B. napus *genetic map, which represented the most gene-rich euchromatic regions distributed across the 10 chromosomes of the A genome. However, this strategy still has limitations since null alleles are very common in *Brassica *in some lines compared with others, as indicated in this and previous studies [26,27]. There may also be situations where the combination of null alleles within complementary paralogous segments in different populations may lead to mis-assignment. Moreover, one may be more confident in the assignment of SSRs where they amplify single polymorphic bands with sizes similar to the expected fragments from DNA derived from amphidiploid plants.

At the time at which this study commenced, 536 seed BACs had been sequenced and released via NCBI-GenBank. Of these, only 161 were assigned to the A genome LGs of *B. napus*, with the remaining 375 unable to be assigned due to the lack of polymorphism amongst the markers developed in this study. Clearly, there would be considerable value to assign all the available sequenced BACs to the *B. napus *LGs. More recently, a total of 1014 sequenced BACs have become available as shown at http://brassica.bbsrc.ac.uk. Even with the imminent availability of sequenced scaffolds from high throughput methods there remains a pressing requirement for alignment with sequenced BACs and unequivocal assignment and orientation to respective chromosomes. Thus, continued development of additional SSR markers from the set of sequenced BACs and sequence scaffolds will facilitate further integration of the A genome LGs in *B. rapa *and *B. napus*, and allow assignment of additional BACs and BAC contigs to the A genome in *B. napus*, especially where these have previously been unassigned. The value of BACs also lies in their ability to be used as probes for chromosome FISH. The mapping of markers derived from previously unassigned sequenced BACs could anchor their source BACs to the A genome in *B. napus*, which then enabled their reliable assignment to the *B. rapa *chromosomes based on the integrity of the A genome between the two species. The integration of the A genome LGs in *B. rapa *and *B. napus *will facilitate the utilization of genetic resources and the rapid transfer of knowledge from *B. rapa *to *B. napus*. Moreover, because the sequenced BACs cover the gene-rich euchromatic regions of the *Arabidopsis *genome, information from the model plant will also assist in understanding the relationships between gene function, genome structure and evolution in *Brassicaceae *and should yield valuable information for rapeseed research [41]. Furthermore, the construction of an integrated genetic map will accelerate the pace of gene mapping and cloning in *B. napus*.

## Conclusions

We developed 890 microsatellite markers from 536 anchored seed BACs and used these to construct a linkage map in *B. napus *integrated with those of *B. rapa*, which included 219 newly developed BrGMS markers. Among these mapped BrGMS loci, 186 were evenly distributed on the A genome LGs. Most of the BrGMS loci on the A genome LGs in *B. napus *are collinear with the homoeologous LGs in *B. rapa*. The mapping of these BAC-specific SSR markers enabled the assignment of 161 *B. rapa *seed BACs, and also their associated BAC contigs to the A genome in *B. napus*. The integrated genetic map will accelerate the exploitation of the *B. rapa *genomic resources for gene tagging and cloning in *B. napus *and for comparative analysis of the A genome of *Brassica *species.

## Methods

### Plant materials

A panel of six *B. napus *breeding lines or cultivars ('S1', 'S2', 'M201', 'M202', 'No2127' and 'ZY821') which were parents of three established mapping populations was used for polymorphism screening of SSR markers [22]. A double-haploid (DH) population, BnaNZDH, with 88 individuals derived from the F1 of a cross between 'No2127' and 'ZY821' [22,42] was used for linkage mapping. Total DNA was isolated from young leaves of the six *B. napus *breeding lines and the 88 DH lines using the cetyltrimethylammonium bromide (CTAB) method [43], and used as template for PCR amplification.

### Source of sequences, microsatellite identification and marker development

The sequences of 536 BACs were obtained from NCBI Genbank and employed for the identification of SSRs. The BAC sequences were submitted to a web-based SSR-discovery tool, SSRPrimer, which integrates the SSR repeat finder Sputnik [44] with the primer design program Primer 3 [45]http://hornbill.cspp.latrobe.edu.au/ssrdiscovery.html, to identify microsatellites. The criteria used for the microsatellite search were a minimum of 6 repeats for di-nucleotide motifs, four repeats for tri-nucleotide motifs and three repeats for tetra- or penta-nucleotide motifs. One to five SSRs with repeat length greater than 20 bp from each BAC were selected for marker development. The parameters for SSR primer design were set as default: primer length from 18 to 23 bp, GC content over 40%, melting temperature between 55°C and 70°C, and PCR products ranging from 100 to 400 bp in size. These newly developed BAC-derived SSR markers were designated as 'BrGMS', representing *Brassica rapa g*enomic *m*icrosatellites. All primers were synthesized by Generay Biotech (Shanghai, China).

All BrGMS markers were used to examine the PCR amplification and polymorphisms using a panel of six rapeseed lines described above. PCR amplification, products separation and staining were performed as described previously [22].

### Linkage analysis and map construction

Polymorphic BrGMS markers were selected to survey the DH lines derived from a cross between 'No.2127' and 'ZY821'. Each DH line was scored as 'A' for the presence of the 'No.2127' allele and 'B' for the presence the 'ZY821' allele for each marker locus. For multiple or duplicated loci detected by a single primer pair, the marker name is suffixed by a lowercase alphabetical letter to distinguish the loci in the order of DNA fragments with increasing length. The linkage map was constructed with JoinMap 3.0 [46]. The Kosambi [47] function was used to convert recombination frequency to map distance (centiMorgan, cM). Most of the linkage groups were determined at LOD scores >6.

## Authors' contributions

JSX carried out genetic mapping, analyzed the data, and drafted the manuscript. XJQ carried out the marker development, participated in genetic mapping, and drafted the manuscript. XFW participated in genetic mapping and the marker development. RYL designed the primers. XMC, YY, SCZ participated in polymorphism detected. JF picked the SSR primers and participated in polymorphism detected. GJK helped draft the manuscript. JSW participated in the design of the study and offered the plant materials. KDL conceived the study, and helped draft the manuscript. All authors read and approved the final manuscript.

## Supplementary Material

Additional file 1 **Additional file 1**. Details of the newly developed SSR markers Click here for file

Additional file 2 **Additional file 2**. The assignments of 125 BACs and their associated contigs to *B. napus *chromosomes. Click here for file

Additional file 3 **Additional file 3**. Assignments of 27 unassigned BACs in current version of physical map in *B. rapa *to *B. napus *linkage map. Click here for file

Additional file 4 **Additional file 4**. Validation of BAC assignments Click here for file

## References

[B1] Labana K Gupta M Importance and origin Monogr Theor Appl Genet 1993 19 1 7

[B2] Paterson A Lan T Amasino R Osborn T Quiros C *Brassica *genomics: a complement to, and early beneficiary of, the *Arabidopsis *sequence Genome Biol 2001 2 1011 1014 10.1186/gb-2001-2-3-reviews1011 PMC138917 11276431

[B3] Lan TH DelMonte TA Reischmann KP Hyman J Kowalski SP McFerson J Kresovich S Paterson AH An EST-enriched comparative map of *Brassica oleracea *and *Arabidopsis thaliana* Genome Res 2000 10 776 788 10.1101/gr.10.6.776 10854410 PMC310908

[B4] Parkin I Gulden S Sharpe A Lukens L Trick M Osborn T Lydiate D Segmental structure of the *Brassica napus *genome based on comparative analysis with *Arabidopsis thaliana* Genetics 2005 171 765 781 10.1534/genetics.105.042093 16020789 PMC1456786

[B5] Lysak M Koch M Pecinka A Schubert I Chromosome triplication found across the tribe *Brassiceae* Genome Res 2005 15 516 525 10.1101/gr.3531105 15781573 PMC1074366

[B6] Yang T Kim J Kwon S Lim K Choi B Kim J Jin M Park J Lim M Kim H Sequence-level analysis of the diploidization process in the triplicated FLOWERING LOCUS C region of *Brassica rapa* Plant Cell 2006 18 1331 1339 10.1105/tpc.106.042572 16632644 PMC1475497

[B7] Yang T Kim J Lim K Kwon S Kim J Jin M Park J Lim M Kim H Kim S The Korea *Brassica *Genome Project: A glimpse of the *Brassica *genome based on comparative genome analysis with *Arabidopsis* Comp Funct Genom 2005 6 138 146 10.1002/cfg.465 PMC2447515 18629219

[B8] Hong C Kwon S Kim J Yang T Park B Lim Y Progress in Understanding and Sequencing the Genome of *Brassica rapa* Int J Plant Genomics 2008 582837 18288250 10.1155/2008/582837 PMC2233773

[B9] Allender CJ King GJ Origins of the amphiploid species *Brassica napus *L. investigated by chloroplast and nuclear molecular markers BMC Plant Biol 2010 10 54 66 10.1186/1471-2229-10-54 20350303 PMC2923528

[B10] UN Genome-analysis in *Brassica *with special reference to the experimental formation of *B. napus *and peculiar mode of fertilization Japan J Bot 1935 7 389 452

[B11] Ayele M Haas BJ Kumar N Wu H Xiao Y Van Aken S Utterback TR Wortman JR White OR Town CD Whole genome shotgun sequencing of *Brassica oleracea *and its application to gene discovery and annotation in *Arabidopsis* Genome Res 2005 15 487 495 10.1101/gr.3176505 15805490 PMC1074363

[B12] Lim Y Plaha P Choi S Uhm T Hong C Bang J Hur Y Toward unraveling the structure of *Brassica rapa *genome Physiol Plant 2006 126 585 591 10.1111/j.1399-3054.2006.00654.x

[B13] Johnston J Pepper A Hall A Chen Z Hodnett G Drabek J Lopez R Price H Evolution of genome size in *Brassicaceae* Ann Bot 2005 95 229 235 10.1093/aob/mci016 15596470 PMC1950721

[B14] Choi S Teakle G Plaha P Kim J Allender C Beynon E Piao Z Soengas P Han T King G The reference genetic linkage map for the multinational *Brassica rapa *genome sequencing project Theor Appl Genet 2007 115 777 792 10.1007/s00122-007-0608-z 17646962

[B15] Kim J Chung T King G Jin M Yang T Jin Y Kim H Park B A sequence-tagged linkage map of *Brassica rapa* Genetics 2006 174 29 39 10.1534/genetics.106.060152 16988107 PMC1569789

[B16] Mun J Kwon S Yang T Kim H Choi B Baek S Kim J Jin M Kim J Lim M The first generation of a BAC-based physical map of *Brassica rapa* BMC Genomics 2008 9 280 290 10.1186/1471-2164-9-280 18549474 PMC2432078

[B17] Morgante M Hanafey M Powell W Microsatellites are preferentially associated with nonrepetitive DNA in plant genomes Nature Genetics 2002 30 194 200 10.1038/ng822 11799393

[B18] Varshney R Graner A Sorrells M Genic microsatellite markers in plants: features and applications Trends in Biotechnology 2005 23 48 55 10.1016/j.tibtech.2004.11.005 15629858

[B19] Gupta P Varshney R The development and use of microsatellite markers for genetic analysis and plant breeding with emphasis on bread wheat Euphytica 2000 113 163 185 10.1023/A:1003910819967

[B20] Burgess B Mountford H Hopkins C Love C Ling A Spangenberg G Edwards D Batley J Identification and characterization of simple sequence repeat (SSR) markers derived in silico from *Brassica oleracea *genome shotgun sequences Mol Ecol Notes 2006 6 1191 1194 10.1111/j.1471-8286.2006.01488.x

[B21] Hong C Piao Z Kang T Batley J Yang T Hur Y Bhak J Park B Edwards D Lim Y Genomic distribution of simple sequence repeats in *Brassica rapa* Mol Cells 2007 23 349 356 17646709

[B22] Cheng X Xu J Xia S Gu J Yang Y Fu J Qian X Zhang S Wu J Liu K Development and genetic mapping of microsatellite markers from genome survey sequences in *Brassica napus* Theor Appl Genet 2009 118 1121 1131 10.1007/s00122-009-0967-8 19190889

[B23] Piquemal J Cinquin E Couton F Rondeau C Seignoret E Doucet I Perret D Villeger M Vincourt P Blanchard P Construction of an oilseed rape (*Brassica napus *L.) genetic map with SSR markers Theor Appl Genet 2005 111 1514 1523 10.1007/s00122-005-0080-6 16187118

[B24] Suwabe K Tsukazaki H Iketani H Hatakeyama K Kondo M Fujimura M Nunome T Fukuoka H Hirai M Matsumoto S Simple sequence repeat-based comparative genomics between *Brassica rapa *and *Arabidopsis thaliana*: the genetic origin of clubroot resistance Genetics 2006 173 309 319 10.1534/genetics.104.038968 16723420 PMC1461432

[B25] Katti M Ranjekar P Gupta V Differential distribution of simple sequence repeats in eukaryotic genome sequences Mol Biol Evol 2001 18 1161 1167 11420357 10.1093/oxfordjournals.molbev.a003903

[B26] Ling A Kaur J Burgess B Hand M Hopkins C Li X Love C Vardy M Walkiewicz M Spangenberg G Edwards D Batley J Characterization of simple sequence repeat markers derived in silico from *Brassica rapa *bacterial artificial chromosome sequences and their application in *Brassica napus* Mol Ecol Notes 2007 7 273 277 10.1111/j.1471-8286.2006.01578.x

[B27] Suwabe K Iketani H Nunome T Kage T Hirai M Isolation and characterization of microsatellites in *Brassica rapa *L Theor Appl Genet 2002 104 1092 1098 10.1007/s00122-002-0875-7 12582617

[B28] Kim H Choi S Bae J Hong C Lee S Hossain M Nguyen D Jin M Park B Bang J Sequenced BAC anchored reference genetic map that reconciles the ten individual chromosomes of *Brassica rapa* BMC Genomics 2009 10 432 446 10.1186/1471-2164-10-432 19751531 PMC2761421

[B29] Lowe A Moule C Trick M Edwards K Efficient large-scale development of microsatellites for marker and mapping applications in *Brassica *crop species Theor Appl Genet 2004 108 1103 1112 10.1007/s00122-003-1522-7 15067397

[B30] Plieske J Struss D Microsatellite markers for genome analysis in *Brassica*. I. Development in *Brassica napus *and abundance in *Brassicaceae *species Theor Appl Genet 2001 102 689 694 10.1007/s001220051698

[B31] Suwabe K Iketani H Nunome T Ohyama A Hirai M Fukuoka H Characteristics of microsatellites in *Brassica rapa *genome and their potential utilization for comparative genomics in *Cruciferae* Breed Sci 2004 54 85 90 10.1270/jsbbs.54.85

[B32] Szewc-McFadden A Kresovich S Bliek S Mitchell S McFerson J Identification of polymorphic, conserved simple sequence repeats (SSRs) in cultivated *Brassica *species Theor Appl Genet 1996 93 534 538 10.1007/BF00417944 24162344

[B33] Iniguez-Luy F Voort A Osborn T Development of a set of public SSR markers derived from genomic sequence of a rapid cycling *Brassica oleracea *L. genotype Theor Appl Genet 2008 117 977 985 10.1007/s00122-008-0837-9 18651126

[B34] Temnykh S DeClerck G Lukashova A Lipovich L Cartinhour S McCouch S Computational and experimental analysis of microsatellites in rice (*Oryza sativa *L.): frequency, length variation, transposon associations, and genetic marker potential Genome Res 2001 11 1441 1452 10.1101/gr.184001 11483586 PMC311097

[B35] Parida SK Yadava DK Mohapatra T Microsatellites in *Brassica *unigenes: relative abundance, marker design, and use in comparative physical mapping and genome analysis Genome 2010 53 55 67 10.1139/G09-084 20130749

[B36] Iniguez-Luy FL Lukens L Farnham MW Amasino RM Osborn TC Development of public immortal mapping populations, molecular markers and linkage maps for rapid cycling *Brassica rapa *and *B. oleracea* Theor Appl Genet 2009 120 31 43 10.1007/s00122-009-1157-4 19784615

[B37] Parkin I Sharpe A Keith D Lydiate D Identification of the A and C genomes of amphidiploid *Brassica napus *(oilseed rape) Genome 1995 38 1122 1131 18470236 10.1139/g95-149

[B38] Szadkowski E Eber F Huteau V Lod M Huneau C Belcram H Coriton O Manzanares-Dauleux M Delourme R King G The first meiosis of resynthesized *Brassica napus*, a genome blender New Phytologist 2010 186 102 112 10.1111/j.1469-8137.2010.03182.x 20149113

[B39] Cheung F Trick M Drou N Lim YP Park JY Kwon SJ Kim JA Scott R Pires JC Paterson AH Comparative analysis between homoeologous genome segments of *Brassica napus *and its progenitor species reveals extensive sequence-level divergence Plant Cell 2009 21 1912 1928 10.1105/tpc.108.060376 19602626 PMC2729604

[B40] Panjabi P Jagannath A Bisht NC Padmaja KL Sharma S Gupta V Pradhan AK Pental D Comparative mapping of *Brassica juncea *and *Arabidopsis thaliana *using Intron Polymorphism (IP) markers: homoeologous relationships, diversification and evolution of the A, B and C *Brassica *genomes BMC genomics 2008 9 113 131 10.1186/1471-2164-9-113 18315867 PMC2277410

[B41] Suwabe K Morgan C Bancroft I Integration of *Brassica *A genome genetic linkage map between *Brassica napus *and *B. rapa* Genome 2008 51 169 176 10.1139/G07-113 18356952

[B42] Xiao S Xu J Li Y Zhang L Shi S Wu J Liu K Generation and mapping of SCAR and CAPS markers linked to the seed coat color gene in *Brassica napus *using a genome-walking technique Genome 2007 50 611 618 10.1139/G07-044 17893738

[B43] Doyle J Doyle J A rapid DNA isolation procedure for small quantities of fresh leaf tissue Phytochem Bull 1987 19 11 15

[B44] The SSR repeat finder Sputnik http://abajian.net/sputnik

[B45] Rozen S Skaletsky H Primer3 on the www for general users and for biologist programmers Methods Mol Biol 2000 132 365 386 10547847 10.1385/1-59259-192-2:365

[B46] Van Ooijen J Voorrips R JoinMap 3.0, Software for the calculation of genetic linkage maps Plant Research International, Wageningen 2001

[B47] Kosambi D The estimation of map distances from recombination values Ann Eugen 1944 12 172 175

